# Different Monoclonal Antibodies in Myasthenia Gravis: A Bayesian Network Meta-Analysis

**DOI:** 10.3389/fphar.2021.790834

**Published:** 2022-01-18

**Authors:** Zhaoming Song, Jie Zhang, Jiahao Meng, Guannan Jiang, Zeya Yan, Yanbo Yang, Zhouqing Chen, Wanchun You, Zhong Wang, Gang Chen

**Affiliations:** ^1^ Department of Neurosurgery and Brain and Nerve Research Laboratory, The First Affiliated Hospital of Soochow University, Suzhou, China; ^2^ Department of Neurosurgery, China-Japan Friendship Hospital, Beijing, China

**Keywords:** monoclonal antibodies, myasthenia gravis, efficacy, safety, meta-analysis

## Abstract

**Background:** Myasthenia gravis (MG) is a common autoimmune disease with acquired neuromuscular transmission disorders. Recently, monoclonal antibodies have been shown to successfully treat a variety of diseases.

**Methods:** In this meta-analysis, an appropriate search strategy was used to search eligible randomized controlled trials (RCTs) on different monoclonal antibodies to treat patients with MG published up to September 2021 from the embase, PubMed, and Cochrane Library. We assessed the average difference or odds ratio between each drug and placebo and summarized them as the average and 95% confidence interval (CI), respectively.

**Results:** In indicators of efficacy, patients receiving eculizumab (MD, −1.9; 95% CI, −3.2–0.76) had decreases in MG-ADL scores compared to placebo. In addition, only eculizumab (MD, −3.1; 95% CI, −4.7–1.5) and efgartigimod (MD, −1.4; 95% CI, −2.1–0.68) showed a significant difference from placebo in the amount of reduction in QMG scores, while neither of the other two monoclonal antibodies was statistically significant. With regard to the safety of monoclonal antibody therapy, there was no significant difference in the probability of AE in subjects treated with any of the four monoclonal antibodies compared to placebo.

**Conclusions:** eculizumab was effective in reducing MG-ADL scores and QMG scores in myasthenia gravis. Meanwhile, eculizumab also caused fewer AE. As an emerging therapy, monoclonal antibodies are prospective in the treatment of MG. However, more researches are required to be invested in the future as the results obtained from small sample sizes are not reliable enough.

## Introduction

Myasthenia gravis (MG) is a common autoimmune disease with acquired neuromuscular transmission disorders (Gilhus and Gravis, 2016). It occurs due to autoantibodies that cause morphological and functional alterations in the postsynaptic membrane, resulting in neuromuscular transmission impairment. The most common pathogenic autoantibody is the acetylcholinesterase receptor (AChR) (Gilhus and Verschuuren, 2015; Evoli, 2017). Approximately 85% of MG patients worldwide are associated with reduced postsynaptic acetylcholine receptor function. Acetylcholinesterase inhibitor has been used in clinical treatment since it was discovered by Walker in 1934. At present, acetylcholinesterase inhibitors and glucocorticoids are recognized as first-line treatments for MG. In recent years, with the improvement of intensive respiratory care and the introduction of immunosuppressive therapy, the mortality of MG patients has been reduced (Grob et al., 2008; Farmakidis et al., 2018; Narayanaswami et al., 2021). However, when patients are treated with glucocorticoid and acetylcholinesterase inhibitors, intolerable adverse reactions or complications often occur, which affect the quality of life (Bae et al., 2006; Konno et al., 2015).

Recently, monoclonal antibodies have been shown to successfully treat a variety of diseases (Alabbad et al., 2020). A series of monoclonal antibodies have also been used in the treatment of MG, such as eculizumab, belimumab, efgartigimod and rozanolixizumab. Eculizumab is a humanized mouse monoclonal antibody that prevents the enzymatic hydrolysis of C5 to C5a and C5b by binding uniquely to C5. Thus, the formation of membrane attack complex (MAC) is prevented and the damage caused by complement fixed AChR antibody is reduced. (Dhillon, 2018; Mastellos et al., 2018). In addition, monoclonal antibodies include efgartigimod and rozanolixizumab, which act on the neonatal Fc receptor (FcRn) target. And belimumab, which acts on the B-cell activating factor of the tumor necrosis factor family (BAFF) target.(Blair and Duggan, 2018; Gable and Guptill, 2019; Mantegazza and Cavalcante, 2019; Alabbad et al., 2020).

Since each monoclonal antibody has different targets and different therapeutic effects, how to select the appropriate monoclonal antibody in clinic is extremely important for the prognosis of patients. (Cai et al., 2019; Albazli et al., 2020). At present, there is no comparative study has been conducted on the treatment of MG with different monoclonal antibodies. In order to prove which monoclonal antibody is more effective and has fewer adverse events in the treatment of MG, we performed a network meta-analysis of multiple monoclonal antibodies and performed a comprehensive comparison and ranking to provide some theoretical evidence to support future clinical treatment.

## Materials and Methods

### Study Protocol

Before we started the research, we drafted a study protocol following the Cochrane Collaboration format. The meta-analysis has not been registered.

### Literature Search

In this study, an appropriate search strategy was used to screen eligible studies on different monoclonal antibodies for the treatment of patients with MG from embase, PubMed and the Cochrane Library. The publication dates were published up to September 2021. The following keyword queries were used: “myasthenia gravis” OR “MG” AND “eculizumab” OR “belimumab” OR “rozanolixizumab” OR “efgartigimod” OR “placebo”.

### Inclusion and Exclusion Criteria

The studies were selected based on the following criteria: 1) randomized controlled trials (RCTs) involving patients with MG receiving monoclonal antibodies; 2) each article must contain at least one outcome variable, such as the Myasthenia Gravis-Activities of Daily Living (MG-ADL) score, the Quantitative Myasthenia Gravis (QMG) score, the incidence of any adverse events (AE) and the incidence of any serious adverse events (SAE); 3) each article must include at least one monoclonal antibody for the treatment of MG, including eculizumab, belimumab, efgartigimod and rozanolixizumab; 4) all subjects must be patients with MG.

### Quality Assessment and Data Extraction

The Cochrane Collaboration risk of bias assessment tool was used to assess the quality of all selected articles. (Higgins et al., 2011). After extraction and identification of eligible articles, two reviewers extracted relevant data for independent assessment, including data on first author, year of publication, study region, duration of follow-up, total number of participants included, population age, and sex ratio. In addition, if disagreements arose during data extraction and quality assessment, conclusions were drawn after discussion with a third reviewer.

### Statistical Analysis

Routine paired meta-analysis and network meta-analysis were performed using Review Manager 5.4.1, R4.0.3 software and gemtc R package according to the Bayesian framework. (van Valkenhoef et al., 2016; Shim et al., 2019). Mean differences (MD) and corresponding 95% confidence intervals (CI) were used as efficient indicators for this analysis. Heterogeneity examinations were firstly applied in the network, and chi-square q-tests and *I* ^*2*^ statistics were used to assess heterogeneity between trials. A random-effects model was used if *p* < 0.05 or *I* ^*2*^ > 50% which showed significant heterogeneity, and a fixed-effects model was used if *p* > 0.05 and *I* ^*2*^ < 50% which showed insignificant heterogeneity. The results of the network meta-analysis contained both direct and indirect comparisons, which were all presented in forest plots. When indirect evidence was present in the data, we analyzed its consistency. To assess the consistency, we compared inconsistencies between direct and indirect sources of evidence. We compared the fitness between the consistency and inconsistency models and compared the differences between direct and indirect evidences, direct and pooled evidences, and indirect and pooled evidences in each closed loop. (van Valkenhoef et al., 2012; White et al., 2012).

In addition, a ranking curve was used to assess the probability of ranking for each outcome indicator. Higher ranking probability values indicate a higher correlation relative to that particular outcome. We estimated the ranking probability for each drug for each outcome and made a line graph of it. The surface under the cumulative ranking curve (SUCRA) was calculated from the treatment level, with a higher SUCRA value indicating a higher rate of outcome occurrence.

## Result

### Study Characteristics

**FIGURE 1:**
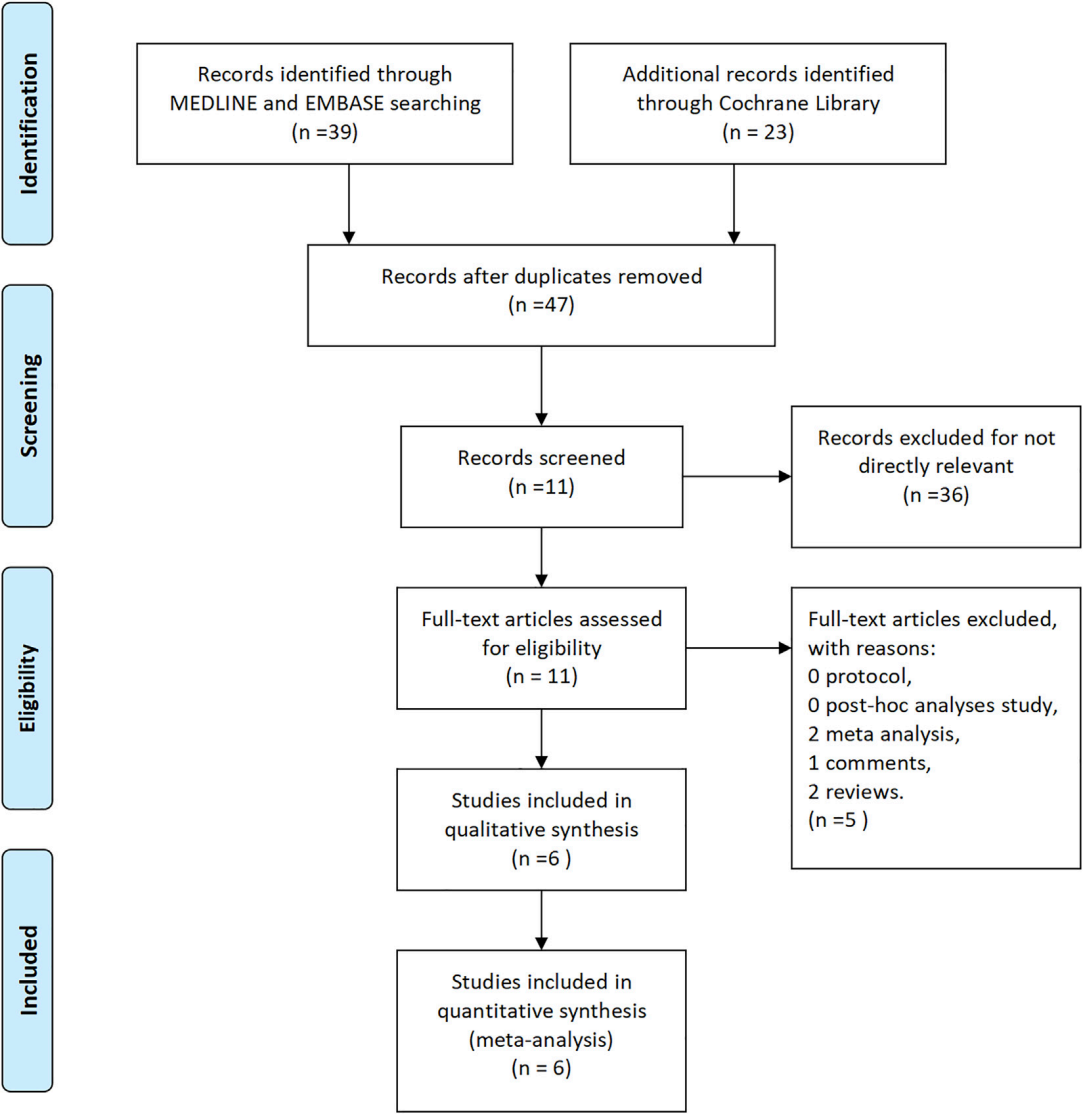
Flow diagram for study identification.

A total of 62 studies were preliminarily retrieved from the literature search according to related keywords. After excluding duplicate studies, 47 studies were left while 15 studies were eliminated. After the review of titles and abstracts, 36 papers were not eligible for inclusion criteria and were excluded. As a result, only 11 articles were included in the network meta-analysis. By analyzing the full text of each article, five articles were finally excluded, including two meta-analyses, one comment, and two reviews. We finally included a total of six articles, including two articles on eculizumab (Howard et al., 2013; Howard et al., 2017), two articles on efgartigimod (Howard et al., 2019; Howard et al., 2021), and one article each on belimumab (Hewett et al., 2018) and rozanolixizumab (Bril et al., 2021). A detailed flow chart of literature screening is shown in Figure 1.

The characteristics of the included studies are listed in Table 1. Specifically, six eligible RCTs, with a total of 412 patients, were included in this network meta-analysis. Among these 412 patients, 69 patients treated with eculizumab, 18 patients treated with belimumab, 96 patients treated with efgartigimod and 21 patients treated with rozanolixizumab. The average age of the participants included in all studies was 48.7 years, and there were more female

**TABLE 1:** Characteristics of the included studies and outcome events.

Study	Countries	Publications	Treatment group, (no of participant)	Diagnosis duration (year)	Female (%)	Mean age±SD (year)	Study period	Outcomes events
Howard et al. (2013)	3	Muscle Nerve	PLA(7) vs ECU(7)	7 ± 7.15	57%	48 ± 10.5	16 weeks	a,b,c,d
Howard et al. (2017)	17	Lancet Neurol	PLA(63) vs ECU(62)	PLA 9.2 ± 8.4	PLA 65%	PLA 47.3 ± 28	26 weeks	a,b,c,d
				ECU 9.9 ± 8.1	ECU 66%	ECU 47.9 ± 25.9		
Hewett et al. (2018)	4	Neurology	PLA(21) vs BEL (18)	PLA 8.30 ± 8.06	PLA 67%	PLA 59.0 ± 13.88	24 weeks	a,b,c,d
				BEL 6.95 ± 9.03	BEL 56%	BEL 52.7 ± 17.32		
Howard et al. (2019)	8	Neurology	PLA(21) vs EFG (12)	PLA 13.3 ± 11.2	PLA 66.7%	PLA 43.5 ± 19.3	80 days	a,b,c,d
				EFG 8.2 ± 9	EFG 53.8%	EFG 55.3 ± 13.6		
Bril et al. (2021)	17	Neurology	PLA(22) vs ROZ (21)	N/A	PLA 64%	PLA 53.3 ± 15.7	100 days	a,b,c,d
					ROZ 62%	ROZ 50.5 ± 14.7		
Howard et al. (2021)	14	Lancet Neurol	PLA(83) vs EFG (84)	N/A	PLA 66%	PLA 48.2 ± 15.0	10 weeks	a,b,c,d
					EFG 75%	EFG 45.9 ± 14.4		

PLA: placebo; ECU: eculizumab; ROZ: rozanolixizumba; EFG: efgartigimod; NLA: not applicant; a:the Myasthenia Gravis Activity of Daily Living(MG-ADL)scale, b:THE, Quantitative Myasthenia Gravis(QMG)scale, c:adverse events, d:serious adverse events.

participants than male participants. The follow-up time in all the included studies was >10 weeks Supplementary Figure S1 shows the established networks for comparison, with each node represents a treatment and the node size and thickness of connections vary according to the number of studies involved in the comparison. In addition, connections between nodes denote direct comparisons.

### Quality Assessments of the Selected Literature

**FIGURE 2:**
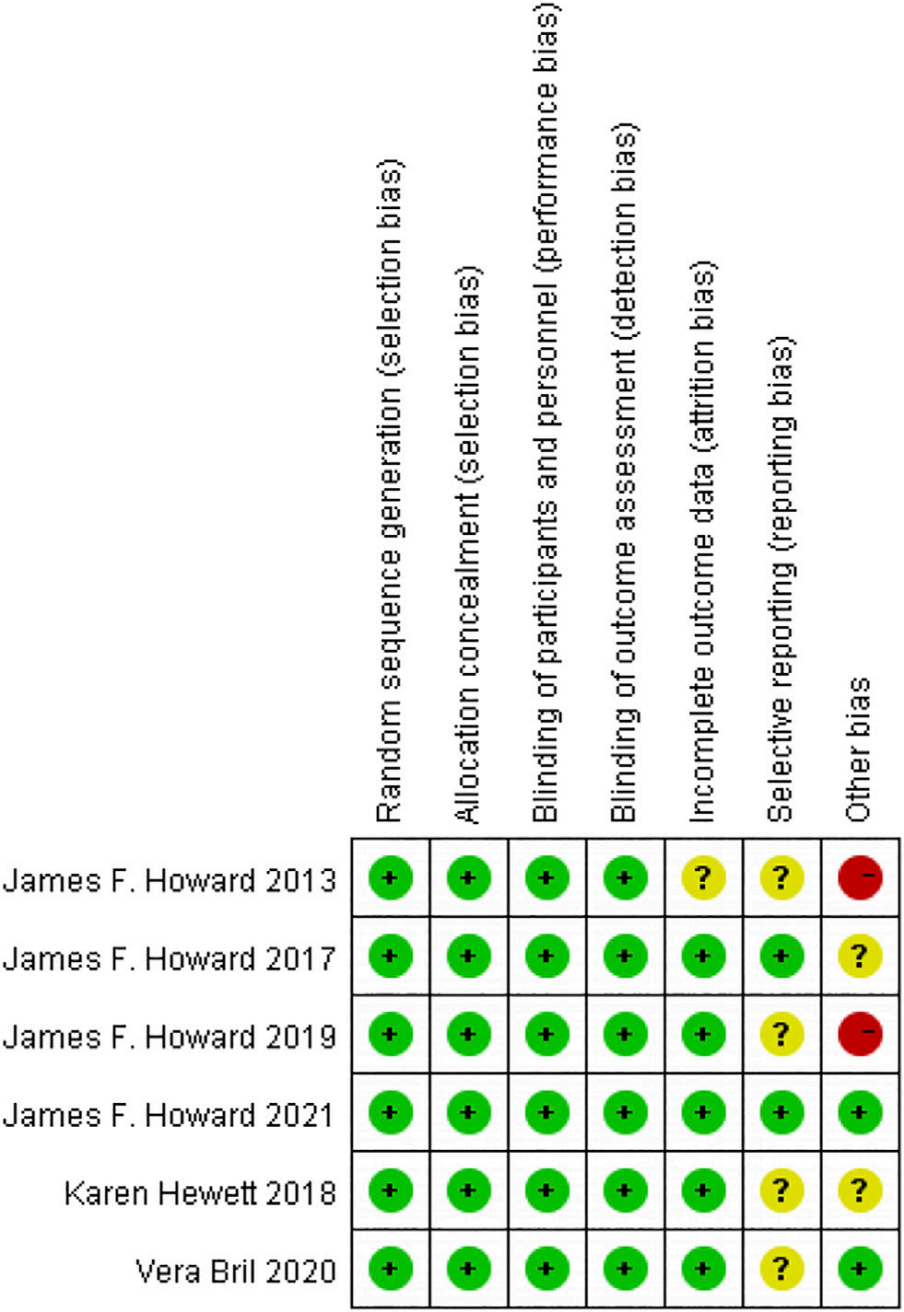
Risks of bias assessment.

The quality assessment of RCTs showed that the overall quality of the publications we included was relatively high, as shown in Figure 2. Although the evaluation results of many articles show that the selective reporting indicators are not clear, only two RCTs have a higher risk of other bias, which is due to the small sample size.

### Network Meta-analysis

We conducted a network meta-analysis to investigate the differences in the efficacy and safety of different monoclonal antibodies for the treatment of patients with MG. The data extracted from each article were summarized and generated as a network graph displayed in the supplementary material (Supplementary Figure S1). In the figure, the circle size corresponds to how much of the population was included and the width of the edge represents how many articles were included between the two pairs. The traces of the network fitting process for each indicator in the gemtc package are shown in the supplementary material (Supplementary Figure S2).

**FIGURE 3:**
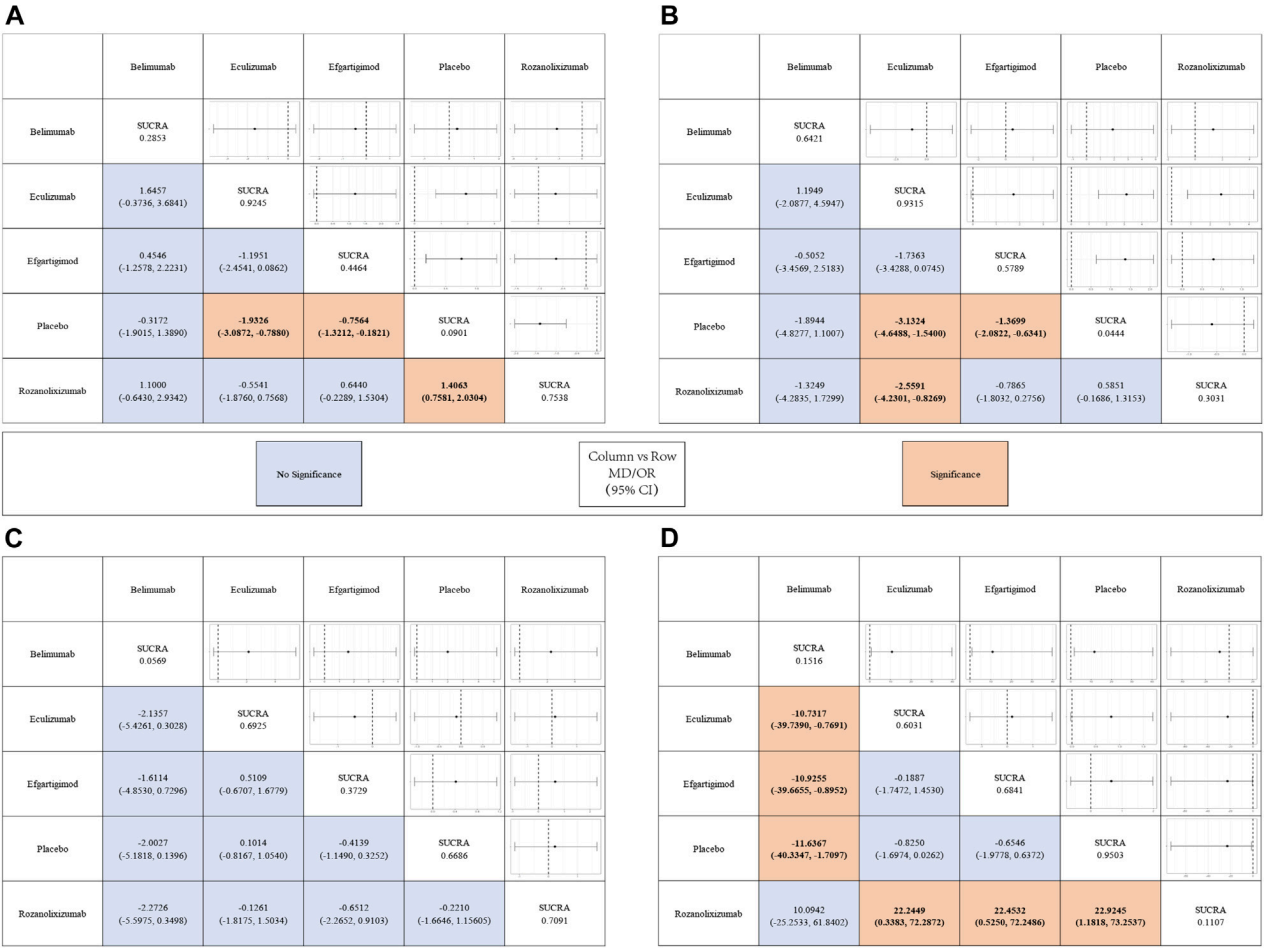
League tables for the outcomes of safety and efficacy generated using the random effects model.

The results simulated according to Markov Monte Carlo method were as follows. In indicators of efficacy, patients receiving eculizumab (MD, −1.9; 95% CI, −3.2–0.76), efgartigimod (MD, −-0.74; 95% CI, −1.3–0.16), and rozanolixizumab (MD, −1.4; 95% CI, −2.1–0.79) had decreases in MG-ADL scores compared to placebo, while belimumab was not significantly different from placebo. And all of the above drugs did not differ statistically significantly from each other when compared in the network. However, only eculizumab (MD, −3.1; 95% CI, −4.7-1.5) and efgartigimod (MD, −1.4; 95% CI, −2.1-0.68) showed a significant difference from placebo in the amount of reduction in QMG scores, while neither of the other two monoclonal antibodies was statistically significant. Interestingly, by network meta-analysis, we found that eculizumab (MD, −2.55; 95% CI, −4.23-0.82) was significantly better than rozanolixizumab in reducing QMG scores (Figure 3).

With regard to the safety of monoclonal antibody therapy, there was no significant difference in the probability of AE in subjects treated with any of the four monoclonal antibodies compared to placebo, nor was there a statistically significant difference between two pairs. However, patients treated with belimumab (OR, 7.0E-8; 95% CI, 7.6E-21-0.15) and rozanolixizumab (OR, 8.4E-11; 95% CI, 9.3E-34-0.17) had a lower risk of SAE compared to those who received placebo, while the other two monoclonal antibodies had no significant difference compared to placebo. Both drugs showed statistically significant differences compared to other drugs in the network. But in the network we did not find a statistically significant difference (belimumab vs rozanolixizumab, OR, 10.0942; 95% CI, −25.2533–61.8402) between these two drugs (Figure 3).

### SUCRA and Rank Probability

**FIGURE 4:**
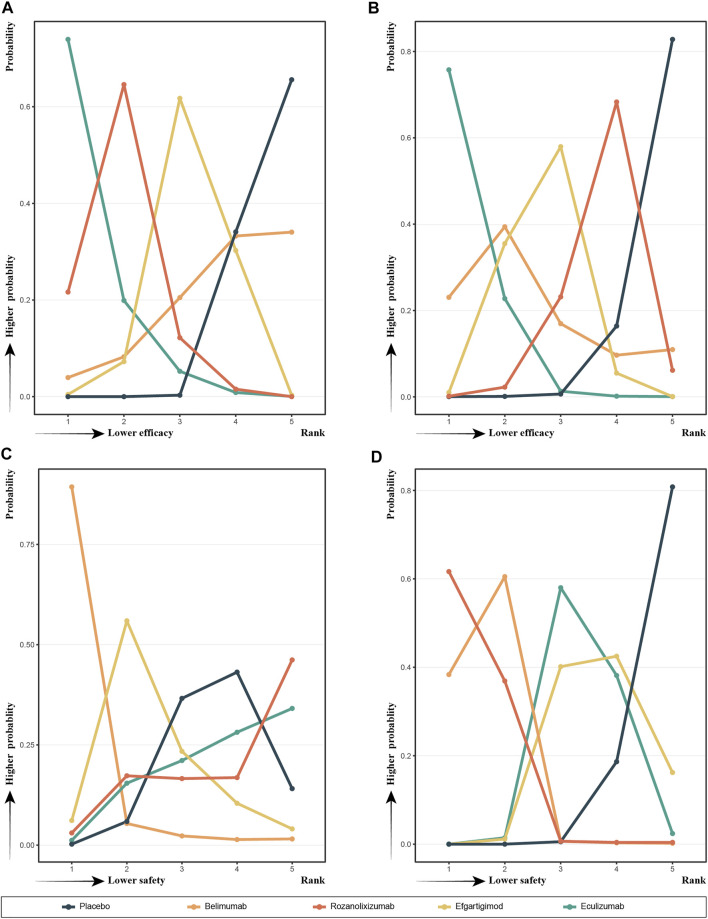
Probability ranks for outcomes of the safety and efficacy generated using the random effects model.

In Figure 4 we show the probability ranking of each treatment strategies corresponding to the different indicators. The line graphs of the probability ranking for each indicator were analyzed together with the SUCRA values in Figure 3.

In terms of efficacy, eculizumab (SUCRA, 0.9245) showed the best improvement in MG-ADL scores, while ronzanolixizumab (SUCRA, 0.7538) showed the second best improvement and efgartigimod (SUCRA, 0.4464) remained better than placebo. Eculizumab (SUCRA, 0.9315) also ranked first in terms of probability of improvement in QMG scores, with belimumab (SUCRA, 0.6421) and efgartigimod (SUCRA, 0.5789) following, and ronzanolixizumab (SUCRA, 0.3031) slightly better than placebo. The above results correspond to the probability ranking results in Figure 4, with the placebo group consistently ranking the worst for efficacy and having a very high probability, while eculizumab had the highest probability of being ranked first for efficacy.

In terms of safety, ronzanolixizumab (SUCRA, 0.7091) ranked highest and with a high probability, indicating that this group was most likely to have AE, with eculizumab (SUCRA, 0.6925) ranking second and placebo (SUCRA, 0.6686) even further higher than efgartigimod (SUCRA, 0.3729) and belimumab (SUCRA, 0.0569). And the incidence of SAE, ronzanolixizumab (SUCRA, 0.1107), belimumab (SUCRA, 0.1516), eculizumab (SUCRA, 0.6031) and efgartigimod (SUCRA, 0.6841) were all probability ranked lower than placebo (Figure 4D).

### Heterogeneity Analysis

**FIGURE 5:**
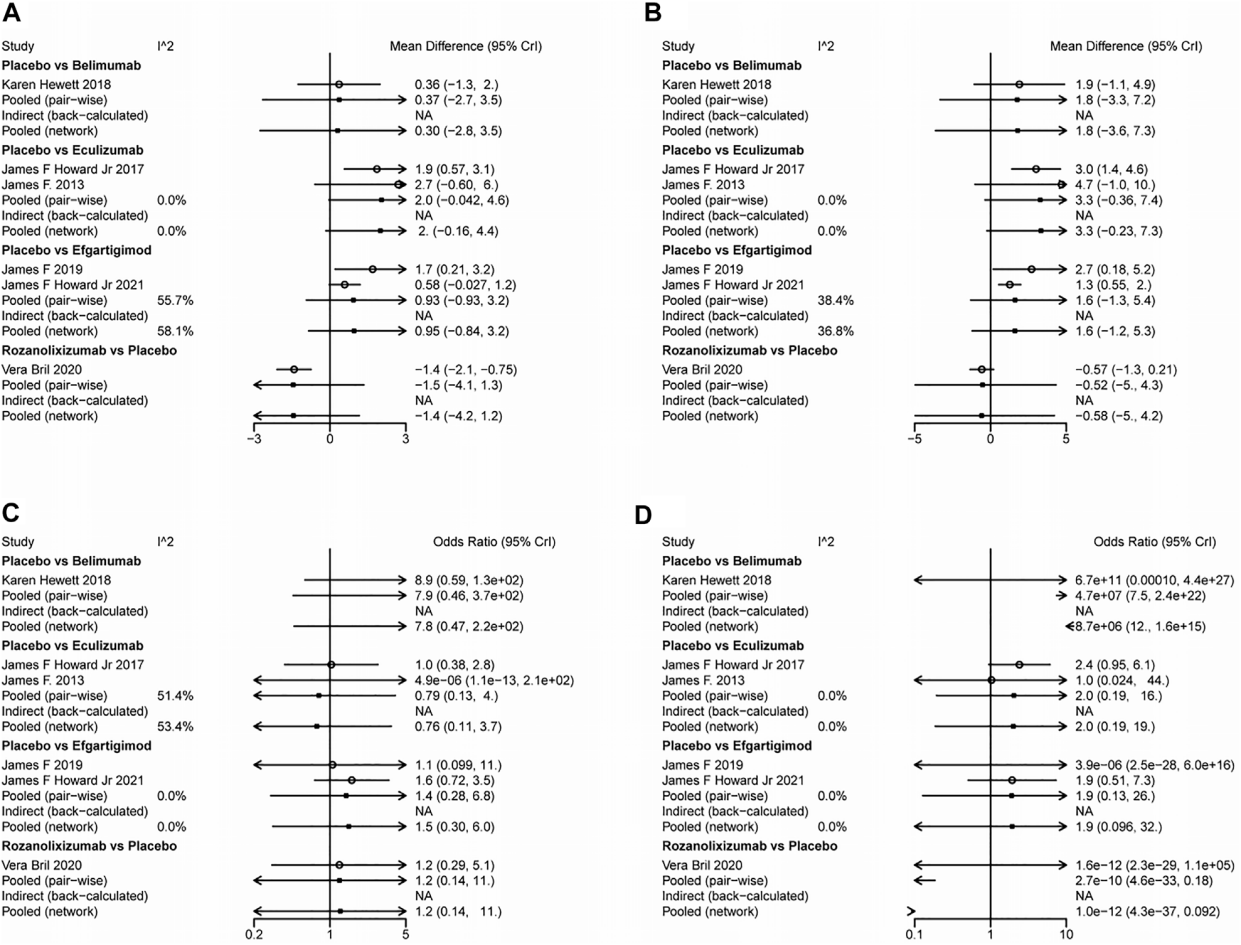
Forest plots for the heterogeneity of efficiency and safety indicators.

We performed heterogeneity analyses of direct, indirect and pooled evidence for each indicator to assess the heterogeneity between each studies included. The I^2^ values of each evidence from different studies are presented in Figure 5. Because the overall I^2^ for each indicator was less than 50%, we chose to use a fixed effects model for all network meta-analyses of MG-ADL (*I* ^*2*^ = 20.24265%), QMG (*I* ^*2*^ = 0%), AE (*I* ^*2*^ = 0.3662987%) and SAE (*I* ^*2*^ = 0%). No indirect evidence of network existence was found in each indicator, so we did not perform a consistency test. Similarity tests were performed by bias analysis and quality control, as belonged to the previous section.

## Discussion

MG is an autoimmune disease, which prevalence is about 32 people per 100,000 population. (Aragonès et al., 2017). This is usually due to AChR antibodies affecting synaptic transmission between neuromuscular junctions. In addition, muscle-specific tyrosine kinase (MuSK) and lipoprotein receptor–related protein 4 (Lrp4) have been found to be involved in the pathogenesis of MG. (El-Salem et al., 2014; Cordts et al., 2017; Gilhus et al., 2019). In the conventional treatment of MG, one can reduce the level of autoantibodies through intravenous immunoglobulin and plasma exchange, and the other can regulate the production of autoantibodies through prednisone and immunosuppressant. (Farmakidis et al., 2018; Sanders et al., 2018). However, these methods are not specific treatments for MG and have many AE. In recent years, with the extensive development of monoclonal antibody technology, the specific modulation of biological pathway has greatly increased the potential therapeutic options. An increasing number of monoclonal antibodies are also used for the treatment of MG, and offer hope to change the therapeutic status of MG. (Dalakas, 2019).

However, no one has studied which monoclonal antibody has the best effect and the least incidence of AE in the treatment of MG. In order to provide some guidance for clinical treatments in the future, we collected and sorted all RCTs on the treatment of MG with monoclonal antibodies published up to September 2021, and performed a network meta-analysis and ranked different drugs. In this study, we chose two efficiency and two safety indicators to evaluate differences between eculizumab, belimumab, efgartigimod and rozanolixizumab treatments in 412 patients with MG.

The two chosen efficiency indicators included QMG scores and MG-ADL scores. The QMG and MG-ADL scores are the most commonly used indicators in clinical treatment. QMG score is a project that needs to be evaluated by doctors, including 13 different items such as ocular, cranial and respiratory. Due to the need for professionals and corresponding equipment, it takes about 20 min to complete QMG scoring. (Howard et al., 2018; McPherson et al., 2020). MG-ADL score, a common questionnaire to evaluate the symptoms and activity status of MG patients, was put forward in the late 1990s. It can be completed within 2–3 min without special training. (Muppidi et al., 2011; de Meel et al., 2019). There are eight questions related to daily life in the questionnaire. Each question has four different scores: 0, 1, 2 and 3. The higher the score, the more serious the patient’s symptoms. MG-ADL is an easy to manage survey of MG, which has a good correlation with QMG and can be used as the secondary efficacy measurement of clinical trials. (Wolfe et al., 1999). In this study, According to our prediction model, we compared the effectiveness of these four drugs. Rozanolixizumab, eculizumab and efgartigimod were all better than placebo in reducing MG-ADL scores, but there was no statistically significant comparison between the drugs. Among the changes of QMG scores, eculizumab and efgartigimod were superior to placebo and eculizumab was superior to rozanolixizumab, but there was no statistically significant difference between the remaining drugs which is same as the previous studys. (Farmakidis et al., 2018; Mantegazza et al., 2020). The effectiveness of each of the four drugs was compared, but it was not evident which drug was most effective in treating MG. We therefore calculated the SUCRA values for each drug and ranked them accordingly. Eculizumab ranked first in improvement of QMG scores, belimumab ranked second, efgartigimod ranked third, and rozanolixizumab ranked last. Among the changes of MG-ADL scores, eculizumab has the best possibility of effect on MG, rozanolixizumab ranked second, efgartigimod ranked in the middle, and belimumab ranked last. As a complement inhibitor, eculizumab could play a therapeutic role by limiting the formation of MAC. (Albazli et al., 2020). eculizumab is also the only monoclonal antibody approved by FAD. Due to the different muscles have different sensitivity to eculizumab, especially eye muscles, QMG will be better than MG-ADL. (de Meel et al., 2018). Efgartigimod and rozanolixizumab can specifically target Fern and inhibit the IgG cycle by prevented the interaction between FcRn and IgG. (Smith et al., 2018). The serum anti-AChR antibody will reduce significantly after the treatment of eculizumab. However, previous studies have proved that there is no association between anti-AChR antibody and clinical improvement (QMG and MG-ADL) of MG. (Sanders et al., 2014). This may explain that the therapeutic effect of efgartigimod and rozanolixizumab is not obvious. Compared with placebo group, belimumab improves indeed in QMG and MG-ADL, but the differences have not statistically significant. This may be due to the fact that some autoantibodies require 1–2 years to decrease significantly after treatment of belimumab. (Ginzler et al., 2014).

The incidence of AE and SAE were chosen as the two indicators to evaluate safety. In our analysis, there were no statistically significant differences between each drug in AE. After calculating the ranking by SUCRA values, we found that belimumab had the lowest possibility of AE, efgartigimod and eculizumab ranked in the middle, while rozanolixizumab had the highest rate. Although the probability of AE in belimumab and efgartigimod was lower than placebo group, the difference between among different drugs was still not statistically significant. In term of SAE, the probability of SAE was lower for belimumab and rozanolixizumab than for efgartigimod, eculizumab and placebo, with no statistically significant differences between the remaining drugs. After ranking, we found that rozanolixizumab had the lowest SAE probability, belimumab ranked second and efgartigimod ranked last. The probability of SAE in each group was lower than that in placebo group, but only belimumab and rozanolixizumab were statistically significant. Rozanolixizumab needs to be used for a long period to achieve better therapeutic effect. The probability of AE may not be high in short-term treatments. (Ginzler et al., 2014). The selectivity of efgartigimod for the IgG binding site of FcRn does not affect the function of FcRn, and thus efgartigimod is unlikely to have a poor safety profile. (Ulrichts et al., 2018). The most common AE after receiving eculizumab treatment is headache and upper respiratory tract infection. Some studies have shown that patients with complement deficiency are prone to life-threatening meningococcal infection. Eculizumab, as a complement inhibitor, often need to be inoculated with meningococcal vaccine in order to prevent this complication. (McNamara et al., 2017). In terms of safety, most monoclonal antibodies are not statistically significant compared with placebo group. We speculate that this is due to the smallsample size and number of studies included in this meta-analysis. In addition, due to the short research time, the occurrence of AE cannot be completely evaluated. Therefore, the results may be limited and need to be further analyzed in the future.

However, our analysis still has some limitations. 1) The number of studies included in this analysis was small, for example, belimumab and rozanolixizumab have only one RCT respectively. There is a certain deviation in our results, which needs a further research. 2) Some RCTs have a small sample size, such as James F. Howard 2013. A small sample size may have a certain impact on the analysis results. 3) Our data and conclusions are based on statistical analysis. Therefore, the clinical effectiveness of this method is not clear, and further research is needed. 4) The study period of some RCTs is only about 10 weeks. The short research period cannot better show the efficiency and safety of treatment, which affects the credibility of the conclusion. 5) In some studies, the duration of MG of the research objects is missing or incomplete, so it is difficult to evaluate whether there are differences between the intervention group and the control group. Thus, there may be a potential impact on the results.

In conclusion, our network meta-analysis of four types of monoclonal antibodies, including eculizumab, belimumab, rozanolixizumab, efgartigimod. Compared the efficiency and safety of different drugs through statistical analysis of six RCTs. Compared with other drugs, eculizumab showed good performance in improving MG-ADL and QMG scores in subjects. However, eculizumab is not much different from placebo in safety indicators. Belimumab and rozanolixizumab could reduce the possibility of SAE. This network meta-analysis provides a theoretical reference for clinical treatments of MG. Due to the limitations we mentioned earlier, this conclusion needs more clinical studies to verify. Overall, monoclonal antibodies are promising as an emerging therapy for the treatment of MG, and more research is still needed.

## Data Availability

The original contributions presented in the study are included in the article/Supplementary Material, further inquiries can be directed to the corresponding authors.

## Abbreviations

CI, confidence interval; MD, mean deviation; MG, Myasthenia gravis; MG-ADL, Myasthenia Gravis-Activities of Daily Living; QMG, Quantitative Myasthenia Gravis score; RCT, randomized controlled trial; RR, risk ratio; SUCRA, surface under the cumulative ranking curve.
