# Supplementary material for: Different Monoclonal Antibodies in Myasthenia Gravis: A Bayesian Network Meta-Analysis
Source: Front Pharmacol. 2022 Jan 18;12:790834. doi: 10.3389/fphar.2021.790834 (PMC8804097; doi:10.3389/fphar.2021.790834)

**Title:** **Different monoclonal antibodies in myasthenia gravis: a Bayesian network meta-analysis**

**Zhaoming Song^1^,#, Jie Zhang^1^,#, Jiahao Meng^1^, Guannan Jiang^1^,** **Zeya Yan^1^, Yanbo Yang^2^, Zhouqing Chen^1^, Wanchun** **You** **^1^,*,** **Zhong Wang ^1^,*, Gang Chen^1^.**

**^1^** ***Department of Neurosurgery & Brain and Nerve Research Laboratory, The First Affiliated Hospital of Soochow University, Suzhou, Jiangsu Province, 215006, China***

**^2^ *Department of Neurosurgery, China-Japan Friendship Hospital, Beijing, 100029, China.***

**Supplement I**

**Network of efficacy and safety indicators. (A) Change in MG-ADL scores. (B) Change in QMG scores. (C) Incidence of any adverse events. (D) Incidence of any serious adverse events.**


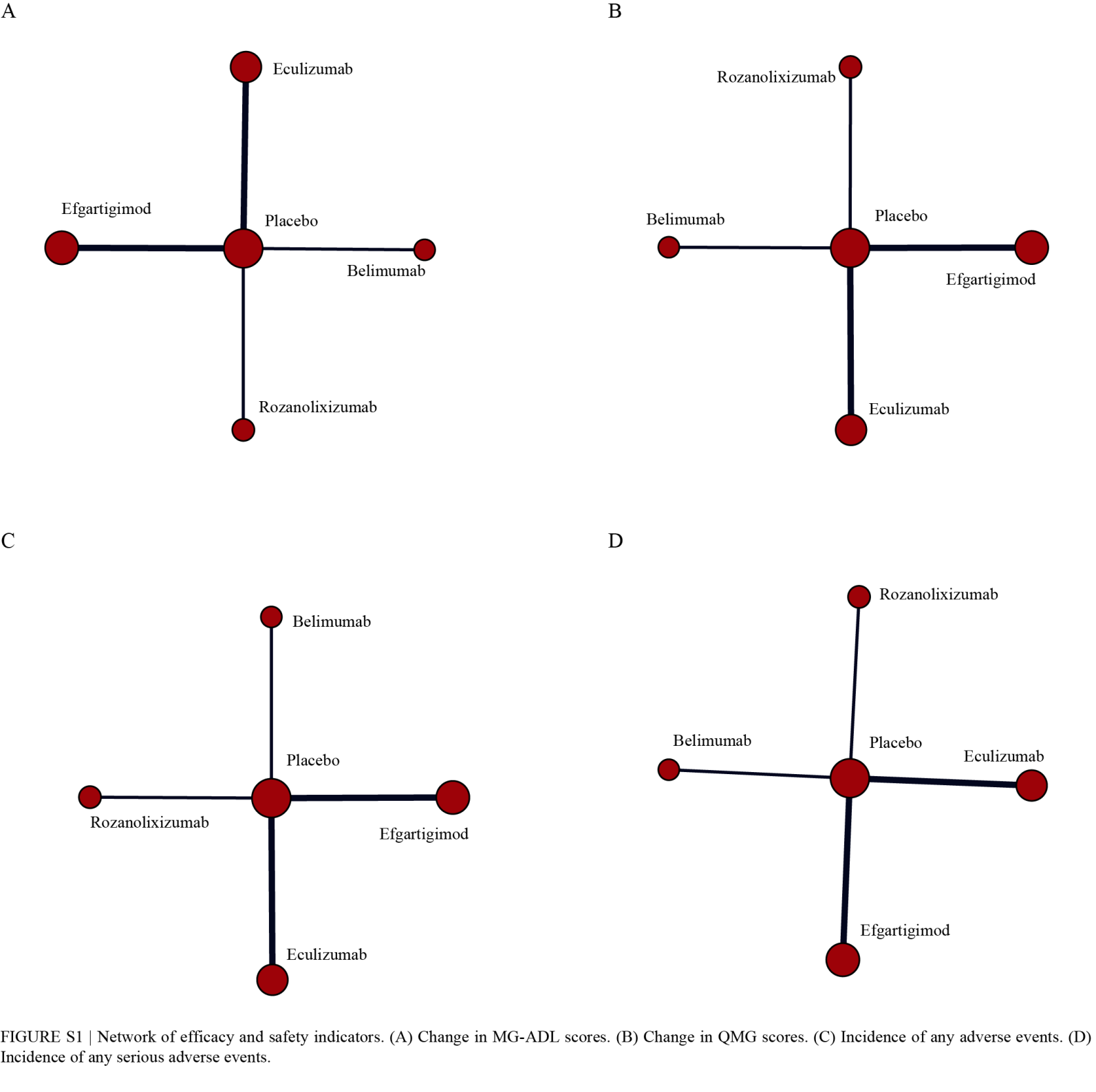


**Supplement II**

**Trace diagram of Markov Monte Carlo method. (A) Change in MG-ADL scores. (B) Change in QMG scores. (C) Incidence of any adverse events. (D) Incidence of any serious adverse events.**


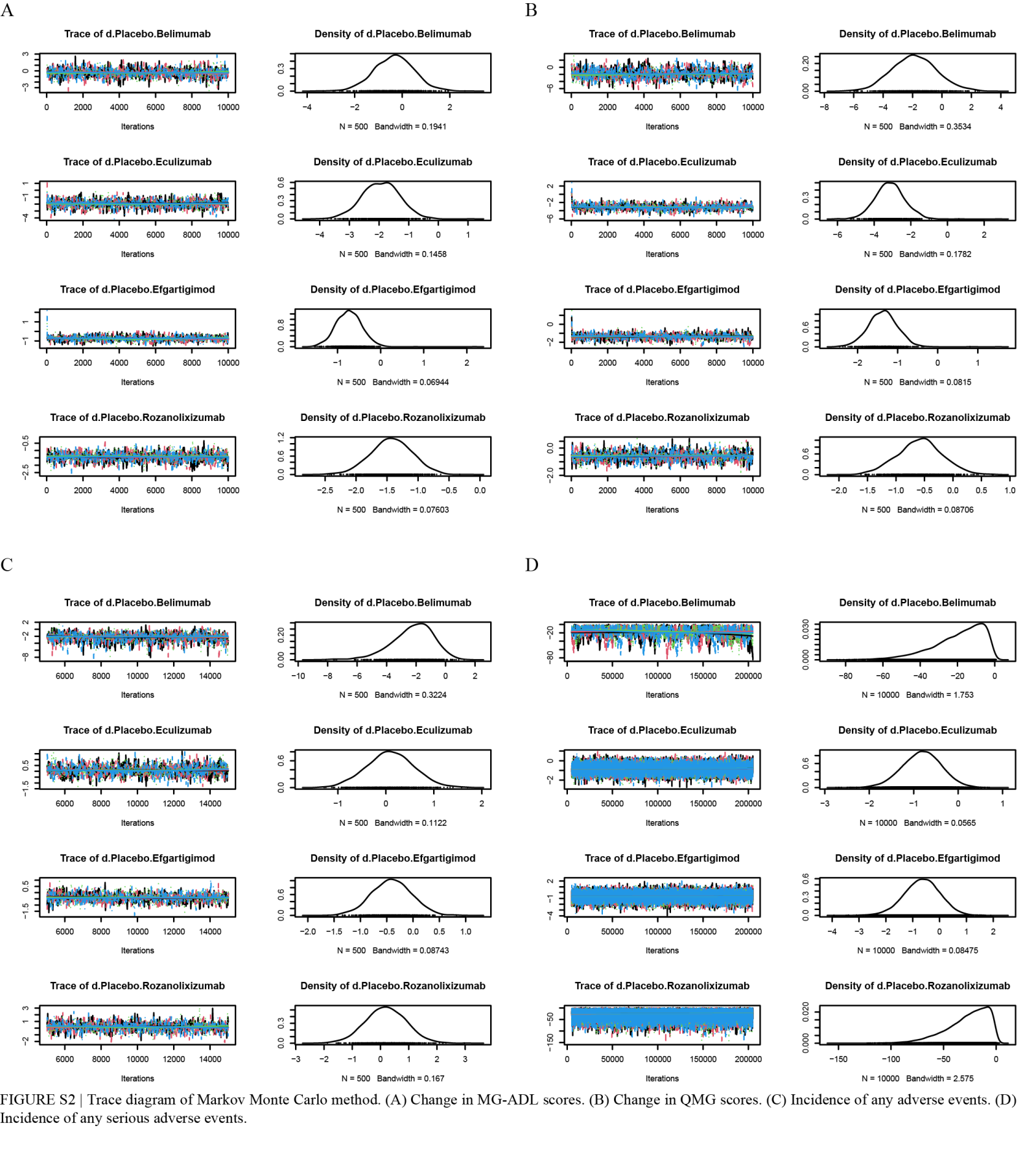

Supplement: Supplementary file 1 [file DataSheet1.docx]
